# Supplementary material for: Fine-Scale Interactions between Leopard Cats and Their Potential Prey with Contrasting Diel Activities in a Livestock-Dominated Nature Reserve
Source: Animals (Basel). 2023 Apr 10;13(8):1296. doi: 10.3390/ani13081296 (PMC10135257; doi:10.3390/ani13081296)
Supplement: Supplementary file 1 [file animals-13-01296-s001.zip › animals-2314081-supplementary.pdf]

Table S1. List of habitat and biotic covariates collected at each camera site for occupancy models in this study

Covariate (abbreviation)	Description	Data source
**Habitat**		
Distance to settlement (DistSettle)	Distance (m) from each camera to the nearest settlement	Calculated from local geographic information dataset
Distance to road (DistRoad)	Distance (m) from each camera to the nearest road	Calculated from local geographic information dataset
Distance to water (DistWater)	Distance (m) from each camera to the nearest supply of water	Calculated from local geographic information dataset
Slope (Slo)	Angle (°) of inclination to the horizontal within 30 m of each camera site	Calculated from local geographic information dataset
Aspect (Asp)	Shady slopes, semi-shady slopes, semi-sunny slopes, or sunny slopes at each camera site	Calculated from local geographic information dataset
Elevation (Ele)	Elevation (m) of the camera sites	Handheld GPS receivers
Enhanced Vegetation Index (EVI)	Vegetation characteristics around each camera site	Derived from a cloud-free Landsat 8 image
Forest cover_100m (Forest100)	Percentage of forest cover around each camera site within a 100 m buffer zone	Derived from 2010 Global Tree Cover database with 30 m resolution
Forest cover_500m (Forest500)	Percentage of forest cover around each camera site within a 500 m buffer zone	Derived from 2010 Global Tree Cover database with 30 m resolution
Forest cover_1000m (Forest1000)	Percentage of forest cover around each camera site within a 1000 m buffer zone	Derived from 2010 Global Tree Cover database with 30 m resolution
**Biotic**		
Leopard_RAI (LeoRAI)	RAI (# photo events per 100 day per camera trap) for leopard	Calculated from records of camera traps
Yellow belly skunk_RAI (YbsRAI)	RAI (# photo events per 100 day per camera trap) for yellow belly skunk	Calculated from records of camera traps
Yellow-throated marten_RAI (YtmRAI)	RAI (# photo events per 100 day per camera trap) for yellow-throated marten	Calculated from records of camera traps
Yellow weasel_RAI (YwRAI)	RAI (# photo events per 100 day per camera trap) for yellow weasel	Calculated from records of camera traps
Dog_RAI (DogRAI)	RAI (# photo events per 100 day per camera trap) for domestic dogs	Calculated from records of camera traps

Table S2. Pearson's correlation coefficients between covariates at the camera site level.

	DistSettle	DistWater	DistRoad	Slo	Asp	Ele	EVI	Forest100	Forest500	Forest1000	LeoEr	YbsRAI	YtmRAI	YwRAI	DogRAI
DistSettle	1.00														
DistWater	0.26	1.00													
DistRoad	0.36	0.03	1.00												
Slo	-0.14	0.08	-0.16	1.00											
Asp	-0.01	0.19	-0.09	-0.06	1										
Ele	0.32	0.37	0.38	0.09	0.12	1.00									
EVI	0.25	0.37	0.27	0.14	-0.08	0.68	1.00								
Forest100	0.38	0.21	0.16	0.02	-0.03	0.33	0.61	1.00							
Forest500	0.55	0.29	0.27	0.01	0.15	0.53	0.57	0.73	1.00						
Forest1000	0.51	0.39	0.27	0.05	0.17	0.68	0.62	0.56	0.89	1.00					
LeoEr	-0.03	0.08	0.23	0.08	0.18	0.43	0.14	0.03	0.07	0.13	1.00				
YbsRAI	0.08	-0.12	0.02	0	0.01	-0.08	0.02	0.16	0.13	0.12	-0.05	1.00			
YtmRAI	-0.12	-0.23	-0.14	-0.09	-0.16	-0.14	-0.33	-0.36	-0.31	-0.29	-0.05	0.01	1.00		
YwRAI	-0.09	-0.07	-0.15	-0.06	0.03	0.03	0.10	0.14	0.08	0.07	0.16	0.37	-0.04	1.00	
DogRAI	0.06	0.15	-0.13	-0.07	-0.01	0.03	0.08	0.18	-0.02	0.02	0.07	-0.13	-0.03	-0.08	1.00

Table S3. The single-species detection models for leopard cats, their prey, and livestock. The top best fitting detection model was carried forward for the next occupancy model.

Species/Model	K	AIC	ΔAIC	W
**Leopard cat**				
p(DistSettle), ψ(.)	3	623.20	0	0.39
p(.), ψ(.)	2	623.59	0.39	0.32
p(DistRoad + DistSettle), ψ(.)	4	624.85	1.65	0.17
p(DistRoad), ψ(.)	3	625.55	2.35	0.12
**Nocturnal rats**				
p(DistRoad + DistSettle), ψ(.)	4	337.46	0	0.97286
p(DistRoad), ψ(.)	3	344.66	7.20	0.02653
p(DistSettle), ψ(.)	3	353.55	16.10	0.00031
p(.), ψ(.)	2	353.62	16.16	0.0003
**Diurnal squirrels**				
p(DistRoad), ψ(.)	3	604.08	0	0.721
p(DistRoad + DistSettle), ψ(.)	4	606.01	1.93	0.2754
p(DistSettle), ψ(.)	3	615.62	11.54	0.0023
p(.), ψ(.)	2	616.59	12.51	0.0014
**Livestock**				
p(DistRoad + DistSettle), ψ(.)	4	633.37	0	8.70e-01
p(DistSettle), ψ(.)	3	637.24	3.87	1.30e-01
p(.), ψ(.)	2	653.80	20.43	3.20e-05
p(DistRoad), ψ(.)	3	655.52	22.15	1.40e-05

Table S4. Summary of top eight single-species occupancy models indicating the role of covariates in determining probability of site use (ψ) and detection (p) of leopard cats, their prey, and livestock.

Species/Model	K	AIC	ΔAIC	W
**Leopard cat**				
p(DistSettle),ψ(DistSettle)	4	620.97	0	0.235
p(DistSettle),ψ(DistWater)	4	621.31	0.34	0.199
p(DistSettle),ψ(DistSettle2)	4	621.44	0.47	0.186
p(DistSettle),ψ(YtmRAI)	4	622.30	1.33	0.121
p(DistSettle),ψ(.)	3	623.20	2.23	0.077
p(DistSettle),ψ(YbsRAI)	4	623.87	2.90	0.055
p(DistSettle),ψ(Ele)	4	624.76	3.79	0.035
p(DistSettle),ψ(LeoRAI)	4	624.87	3.90	0.034
**Nocturnal rats**				
p(DistRoad + DistSettle), ψ(Asp)	5	330.73	0	0.638
p(DistRoad + DistSettle), ψ(EVI)	5	335.01	4.28	0.075
p(DistRoad + DistSettle), ψ(Forest100)	5	335.58	4.85	0.056
p(DistRoad + DistSettle), ψ(Ele2)	5	336.17	5.44	0.042
p(DistRoad + DistSettle), ψ(Ele)	5	336.29	5.56	0.040
p(DistRoad + DistSettle), ψ(Forest1000)	5	336.94	6.21	0.029
p(DistRoad + DistSettle), ψ(DistRoad)	5	337.12	6.39	0.026
p(DistRoad + DistSettle), ψ(.)	4	337.46	6.73	0.022
**Diurnal squirrels**				
p(DistRoad), ψ(Forest100)	4	604.08	0	0.120
p(DistRoad), ψ(.)	3	604.08	0.01	0.120
p(DistRoad), ψ(Asp)	4	604.75	0.67	0.086
p(DistRoad), ψ(Slo)	4	604.76	0.68	0.085
p(DistRoad), ψ(Ele)	4	605.17	1.09	0.070
p(DistRoad), ψ(Forest1000)	4	605.23	1.14	0.068
p(DistRoad), ψ(Forest500)	4	605.23	1.14	0.068
p(DistRoad), ψ(Ele^2)	4	605.54	1.46	0.058
**Livestock**				
p(DistRoad + DistSettle), ψ(.)	4	633.37	0	0.123
p(DistRoad + DistSettle), ψ($DistSettle^2$)	5	633.44	0.07	0.119
p(DistRoad + DistSettle), ψ(Asp)	5	633.87	0.50	0.096
p(DistRoad + DistSettle), ψ(Forest1000)	5	634.00	0.63	0.090
p(DistRoad + DistSettle), ψ(DistSettle)	5	634.50	1.13	0.070
p(DistRoad + DistSettle), ψ(Slo)	5	634.61	1.24	0.066
p(DistRoad + DistSettle), ψ(DistWater)	5	634.99	1.62	0.055
p(DistRoad + DistSettle), ψ(DistRoad)	5	635.04	1.67	0.053

Table S5. Regression coefficients from the SEM model for leopard cat, their potential prey and livestock in higher grazing sites. The χ^2 test statistic was used, with 1 degree of freedom, and *P* value was 0.590, which indicated moderate to good fit.

**Regressions (direct effect)**:			Estimate	SE	Z-Score	*P* value
Leopard cat	~	Livestock	0.124	0.146	0.849	0.396
nocturnal rats	~	Livestock	-0.163	0.183	-0.893	0.372
Diurnal squirrels	~	Livestock	-0.079	0.188	-0.420	0.675
Leopard cats	~	Nocturnal rats	0.632	0.117	5.423	0.000
Leopard cats	~	Diurnal squirrels	0.148	0.145	1.024	0.306
**Defined Parameters (indirect effect)**:						
Effect of Livestock on Leopard cats through diurnal squirrels			-0.010	0.026	-0.376	0.707
Effect of Livestock on Leopard cats through nocturnal rats			-0.020	0.033	-0.613	0.540
Total effect of Livestock on Leopard cats			0.094	0.115	0.815	0.415

Table S6. Regression coefficients from the SEM model for leopard cats, their potential prey and livestock in lower grazing sites. The χ^2 test statistic was used, with 1 degree of freedom, and *P* value was 0.674, which indicated moderate to good fit.

**Regressions (direct effect)**:			Estimate	SE	Z-Score	*P* value
Leopard cats	~	Livestock	-0.080	0.165	-0.484	0.629
Nocturnal rats	~	Livestock	0.119	0.186	0.643	0.520
Diurnal squirrels	~	Livestock	0.385	0.155	2.484	0.013
Leopard cats	~	Nocturnal rats	0.526	0.133	3.944	0.000
Leopard cats	~	Diurnal squirrels	-0.280	0.161	-1.742	0.082
**Defined Parameters (indirect effect)**:						
Effect of Livestock on Leopard cats through diurnal squirrels			-0.031	0.065	-0.475	0.635
Effect of Livestock on Leopard cats through nocturnal rats			-0.010	0.025	-0.385	0.700
Total effect of Livestock on Leopard cats			-0.120	0.249	-0.482	0.630

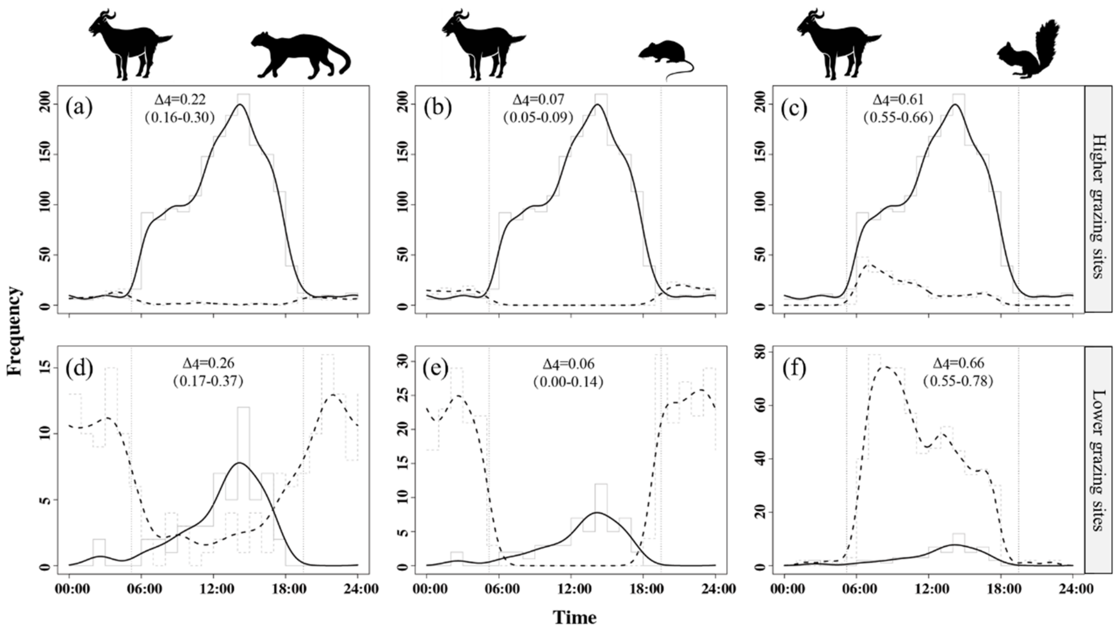

Figure S1. Pairwise comparisons of daily activity patterns between the livestock (solid line) and leopard cats as well as their potential prey (broken lines) in higher and lower grazing sites. Overlap coefficients (Δ), their respective 95% confident intervals are shown at the top of each graph. The vertical black dashed lines in x-axis represent the sunrise the sunset.

Table S7. Proportion of detections in each period of leopard cats and diurnal squirrels in higher and lower grazing sites.

Species	Dawn	Day	Dusk	Night
Leopard cats				
Higher grazing sites	12.15 %	13.08 %	8.41 %	66.36 %
Lower grazing sites	10.53 %	15.79 %	8.55 %	65.13 %
Diurnal squirrels				
Higher grazing sites	3.80%	94.09%	2.11 %	
Lower grazing sites	1.94%	95.63%	2.43 %	
